# The nonlinear effect of time pressure on innovation performance: New insights from a meta-analysis and an empirical study

**DOI:** 10.3389/fpsyg.2022.1049174

**Published:** 2023-01-09

**Authors:** Haojie Song, Renjing Gao, Qiang Zhang, Yongxin Li

**Affiliations:** ^1^School of Business, Zhengzhou University, Zhengzhou, Henan, China; ^2^Shenzhen Health Development Research and Data Management Center, Shenzhen, China; ^3^Department of Health Policy Research, Henan University, Kaifeng, Henan, China

**Keywords:** time pressure, innovation performance, learning behavior, supervisor developmental feedback, meta-analysis

## Abstract

As competition grows, when employees are required to accelerate innovation, they also face increasing time pressure. In order to shed light on how time pressure affects employees’ innovation performance, two studies were conducted to examine the effect of time pressure on innovation performance. In Study 1, based on 50 effect sizes from 50 independent samples (*N* = 15,751) in 40 articles, a meta-analysis was conducted to examine the J-shaped effect of time pressure on innovation performance. In Study 2, based on a two-wave survey of 645 employees, the mechanism underlying the J-shaped effect of time pressure on innovation performance was explored. Results from Study 1 revealed that time pressure had a J-shaped effect on innovation performance, such that high levels of time pressure had a more positive effect on innovation performance. Results from Study 2 showed that learning behavior significantly mediated the J-shaped effect of time pressure on innovation performance, and that supervisor developmental feedback moderated the intermediary process. These results deepen the understanding of the relationship between time pressure and innovation performance, and provide practical advice on how to manage innovation performance under time pressure.

## Introduction

As the backbone of innovation productivity, employees’ innovation performance is directly related to the development of organizations (Zhong et al., 2018). Employees’ innovation performance refers to the level of employee involvement in innovation, which is associated with consciously creating, introducing, and applying new methods to achieve innovative work, such as problem identification, generating new ideas, and proposing original solutions (Li and Wang, 2021). With increasing competition, innovation has become a central objective of organizational development, and many organizations require employees to innovate at a faster pace, with a greater focus on accelerating innovation and compressing timelines (Yao et al., 2020). Thus, when employees are required to accelerate innovation, they also face increasing time pressure. The phenomenon has attracted the attention of many researchers, and existing studies have come to various conclusions about the relationship between time pressure and innovation performance. For example, based on the cognitive appraisal theory of stress, some studies have suggested that employees might evaluate time pressure as a challenging stressor, leading to a positive effect of time pressure on innovation performance (Adler and Koch, 2017; Bormann, 2020). However, according to the self-determination theory, some studies found that time pressure had a negative effect on innovation performance by impairing employees’ perception of self-determination (Naotunna and Zhou, 2021). Following the ground-breaking theory of creative thought, some studies suggested that if employees could not think through problems fully under time pressure, this might limit innovative thinking and then reduce innovation performance (Amabile et al., 2002). Finally, guided by the activation theory, some studies found that there was an inverted U-shaped relationship between time pressure and innovation performance, in which only a medium level of time pressure could maximize innovation performance (Baer and Oldham, 2006; Ohly et al., 2006; Binnewies and Wornlein, 2011).

Previous studies have laid a foundation for comprehending the relationship between time pressure and innovation performance. However, the relationship between time pressure and innovation performance remains unclear. Although a few studies have suggested that there might be a J-shaped relationship between time pressure and innovation performance (Aleksic et al., 2017), there was neither the systematic theoretical analysis nor the empirical research necessary to support and test this viewpoint. The attentional focus model suggests that employees may generate a higher level of attention under high pressure (Johnson et al., 2019), thus enhancing both convergent and divergent thinking (the two dominant components of creativity; Kousoulas, 2010; Zhang et al., 2020). If so, medium and higher levels of time pressure may not be a turning point at which innovation performance begins to decline, but rather a turning point at which innovation performance begins to rise sharply. Therefore, in order to shed more light on the relationship, this paper aims to explore the J-shaped effect of time pressure on innovation performance.

Previous studies have mainly analyzed employees’ passive emotional and cognitive responses in the face of time pressure based on the cognitive appraisal theory of stress, self-determination theory, etc., and have rarely explored employees’ active learning behavior under time pressure. However, guided by the active learning hypothesis, when job demands exceed the resources of individuals, they may perceive higher job demands and stronger threats, and then actively generate learning behaviors to obtain more resources to cope with stress (Karasek and Theorell, 1990; Daniels et al., 2009). Further, given that learning behavior is not only a main way to cope with stress (Andre et al., 2022) but also an important path for improving innovation performance (Holman et al., 2012), learning behavior may play a mediating role in the J-shaped relationship between time pressure and innovation performance. Therefore, it is essential to identify the mediating effect of learning behavior in order to bridge this gap.

Finally, existing studies have mainly discussed the moderating effects of individual characteristics on the relationship between time pressure and innovation performance, such as dispositional mindfulness, neuroticism, time management skills, etc. (Antwi et al., 2019; Rostami et al., 2019; Bormann, 2020), and have rarely explored the boundaries from the perspective of leader behavior. However, there are frequent interactions between employees and their supervisors, and the developmental feedback provided by supervisors can provide employees with sufficient social support to cope with stress (Fang et al., 2021). The active learning hypothesis of the job demands-control-support (JDCS) model states that social support may affect an individual’s sense of control in the face of higher job demands, and then moderate the relationship between higher job demands and learning behavior (Ouweneel et al., 2009; Goner et al., 2020). Therefore, based on the active learning hypothesis, supervisor developmental feedback may have a significant moderating effect on the relationship between time pressure and learning behavior, and then moderate the indirect effect of time pressure on innovation performance through learning behavior. It is essential to expand existing literature by exploring the moderating effect of supervisor developmental feedback on the relationship between time pressure and innovation performance.

To summarize, based on the attentional focus model and the active learning hypothesis, we aim to examine the J-shaped effect of time pressure on innovation performance and its underlying mechanism, including the mediating effect of learning behavior in the J-shaped relationship between time pressure and innovation performance, and the moderating effect of supervisor developmental feedback in the intermediary process.

## Theory and hypotheses

### The J-shaped effect of time pressure on innovation performance

The attentional focus model states that a lack of time resources will affect an individual’s attention to the object and scope in the task environment and limit attention to task-related factors (Karau and Kelly, 1992). Based on the attentional focus model, individuals under high time pressure exhibit more task-focused attention, while non-task-focused attention occurs if abundant time is available (Li et al., 2015). Specifically, threatened people under high time pressure are highly motivated to focus their attention and devote their cognitive resources to manage the threat at hand. Compared to low-imminent threats, high-imminent threats would increase people’s attention and cognitive resources due to their heightened motivation to resolve the threatening situation (Cheng et al., 2018; Johnson et al., 2019).

Following the attentional focus model, because time pressure is a perception of not having adequate time to complete a task, once time pressure rises to a higher level and causes employees to have a stronger threat perception, they will devote their full attention to the task. Furthermore, given that continuous and highly concentrated attention is conducive to the generation of convergent thinking and improving innovation performance (Mekern et al., 2019), there may be a dynamic nonlinear relationship between time pressure and innovation performance. That is, when time pressure is at a lower level and employees have a lower sense of threat, their attention is relatively distracted, and then innovation performance is not significantly improved. But once time pressure rises to the medium and higher levels, causing employees to have a stronger sense of threat, they will focus all their attention on work and strengthen their convergent thinking to carry out in-depth information processing, which is conducive to the generation of new problem-solving ideas (Kim et al., 2010), thus leading to an improvement in innovation performance. Therefore, time pressure may have a J-shaped effect on innovation performance. At the beginning, the effect of time pressure on innovation performance may not be significant, but when a higher level of time pressure level is reached, the effect becomes significantly positive. In summary, the following hypothesis is proposed:

*H1*: Time pressure has a J-shaped effect on innovation performance: compared with lower time pressure, higher time pressure has a more significantly positive effect on innovation performance.

### The mediating effect of learning behavior

Learning behavior is the means by which employees acquire new knowledge and new skills from external resources (Zhang and Liang, 2021). According to the active learning hypothesis, it is a main way for individuals to deal with job demands, through which they can gain new knowledge and find more information to cope with job demands (Karasek and Theorell, 1990; Daniels et al., 2009). The active learning hypothesis states that when job demands are high, employees must integrate new knowledge or skills through learning behavior to perform tasks that cannot be solved alone (Wielenga-Meijer et al., 2010). Specifically, when external job demands exceed individuals’ own resources, they may perceive stronger threats, then actively generate learning behaviors to acquire more resources to cope with the stress; and the stronger the threat, the more learning willingness and learning behavior are generated (Hausser et al., 2014; Decius et al., 2021). Based on the active learning hypothesis, when employees are under lower time pressure, they may be able to rely on their own resources to cope with the demands of the job, and thus experience a weaker sense of threat, which will not stimulate learning behaviors. However, once time pressure rises to a higher level and causes employees to think that their own resources are insufficient to cope with demands, they may experience a stronger sense of threat, and engage a lot of learning behaviors—for example, consulting supervisors, communicating with colleagues and participating in training, all of which can help them obtain more resources to deal with the higher demands of the job. In summary, the following hypothesis can be put forward:

*H2*: Time pressure has a J-shaped effect on learning behavior: compared with lower time pressure, higher time pressure has a stronger positive effect on learning behavior.

Learning behavior can further promote innovation performance. Firstly, learning behavior can help employees obtain new perspectives and methods, which can expand their divergent thinking, and thus help them explore new ideas and pathways to deal with their current work problem (Kousoulas, 2010). Secondly, given that learning behavior is a process of continuous accumulation of knowledge (Wang et al., 2021), and an abundance of knowledge is conducive to the generation of innovative ideas (Gong et al., 2009), it is helpful for employees to break through previous thinking inertia and propose creative solutions through learning behavior. Finally, employees can resist uncertainty and enhance their confidence in dealing with innovation risks by learning new knowledge (Lankau and Scandura, 2002), and then dare to try out new and different creative ideas. In summary, the following hypothesis is proposed:

*H3*: Learning behavior has a positive effect on innovation performance.

As mentioned above, based on hypotheses 2 and 3, because time pressure has a J-shaped effect on learning behavior, and learning behavior has a positive effect on innovation performance, the following hypothesis can be proposed:

*H4*: Time pressure has a J-shaped indirect effect on innovation performance through the mediating effect of learning behavior: compared with lower time pressure, higher time pressure has a more significantly positive effect on innovation performance through learning behavior.

### The moderating role of supervisor developmental feedback

According to the active learning hypothesis, not only do higher job demands lead to learning behaviors, but also this relationship is moderated by social support (Daniels et al., 2009). Social support refers to the support individuals obtain from social relationships such as colleagues and supervisors, manifesting in assistance in problem-solving situations. Based on the active learning hypothesis, social support may affect an individual’s sense of control in the face of higher job demands, and then moderate the relationship between higher job demands and learning behavior (Goner et al., 2020). Specifically, if employees perceive a higher level of social support, they can rely on others when facing job demands that cannot be solved alone. In this condition, employees may engage in more learning behaviors when they are dealing with higher job demands, given that higher levels of social support can increase employees’ controlled perception of the higher job demands. Otherwise, learning behaviors may become less and less as the job demands gradually increase, given that lower levels of social support can cause employees to see higher job demands as an uncontrolled stressor, which would discourage learning behaviors (Ouweneel et al., 2009).

Following the active learning hypothesis, supervisor developmental feedback may moderate the effect of time pressure on learning behavior. Supervisor developmental feedback refers to constructive information provided by supervisors (Zhou, 2003). Unlike traditional feedback, which focuses on evaluating task performance, supervisor developmental feedback emphasizes that supervisors share not only their own task-related knowledge and experience with subordinates, but also impart information useful for individual growth and development to employees (Zhou, 1998; Guo et al., 2020). In addition, supervisors convey trust and clarify expectations to employees, which can effectively alleviate employees’ negative stress reactions (Li et al., 2011). As mentioned above, supervisor developmental feedback can provide employees with sufficient social support to cope with stress. Therefore, based on the active learning hypothesis, it is possible that supervisor developmental feedback may play a significant moderating role in the relationship between time pressure and learning behavior, given that supervisor developmental feedback can provide employees with sufficient social support to cope with stress, thereby increasing employees’ controlled perception of the higher job demand.

Specifically, when the level of supervisor developmental feedback is higher, time pressure may have a J-shaped effect on learning behavior. In this condition, employees can obtain sufficient social support. Even if the time pressure rises to a level that makes employees feel the stronger threat of higher job demands, employees can still cope with the pressure by enhancing their ability and confidence through supervisor developmental feedback, which will make employees see higher time pressure as a controlled stressor, generating more learning behaviors. Conversely, when the level of supervisor developmental feedback is lower, time pressure may have an inverted J-shaped effect on learning behavior. In this condition, because employees receive relatively little social support from their supervisors, they have difficulty coping with stress. Once the time pressure rises to a certain level, employees may feel isolated, helpless, and frustrated, which will cause them to see higher time pressure as an uncontrolled stressor that discourages learning behaviors. In summary, the following hypothesis is proposed:

*H5*: Supervisor developmental feedback moderates the J-shaped effect of time pressure on learning behavior: when the level of supervisor developmental feedback is higher, time pressure has a J-shaped effect on learning behavior; otherwise, there is an inverted J-shaped effect.

As mentioned above, based on hypotheses 4 and 5, because time pressure has a J-shaped effect on innovation performance through the mediating effect of learning behavior, and supervisor developmental feedback moderates the relationship between time pressure and learning behavior, the following moderated mediation hypothesis is proposed:

*H6*: Supervisor developmental feedback moderates the J-shaped effect of time pressure on innovation performance through learning behavior: when the level of supervisor developmental feedback is higher, time pressure has a J-shaped indirect effect on innovation performance through learning behavior; conversely, when the level of supervisor developmental feedback is lower, time pressure has an inverted J-shaped indirect effect on innovation performance through learning behavior.

**Figure 1:**
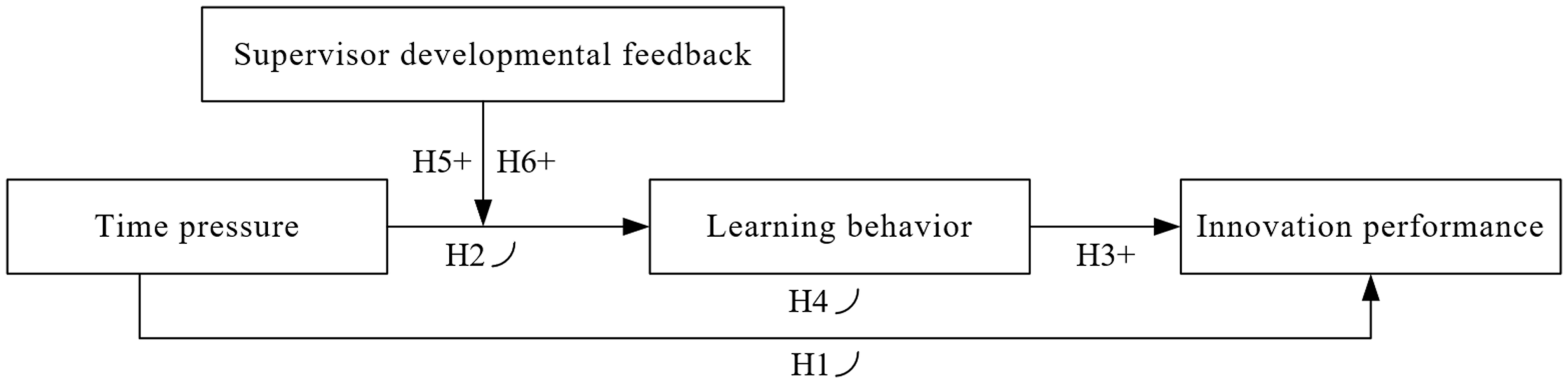
Research conceptual model.

To summarize, we propose a theoretical model as shown in Figure 1. We will test the theoretical model through two studies: Study 1 uses meta-analysis to test the J-shaped relationship between time pressure and innovation performance (*H1*); Study 2 uses a two-wave survey to explore the internal mechanism underlying the J-shaped effect, including the mediating effect of learning behavior and the moderating effect of supervisor developmental feedback (*H1*–*H6*).

## Study 1

### Materials and methods

#### Literature search and inclusion criteria

We began our literature search by searching for subject terms including time pressure, time stress, time crunch, time poverty, creativity, innovative behavior, innovation performance, creative behaviors, and creative performance. Using these terms, we conducted an extensive literature search in the following databases: CNKI, VIP, Wanfang, Web of Science, Wiley, EBSCO-Academic Search Premier, ProQuest, ABI/Inform, PsycINFO, and Google Scholar. Finally, we also searched important journals in the field of management and psychology, and reviewed the references from identified literature for additional possible literature. The deadline for literature research is February 2022.

**Figure 2:**
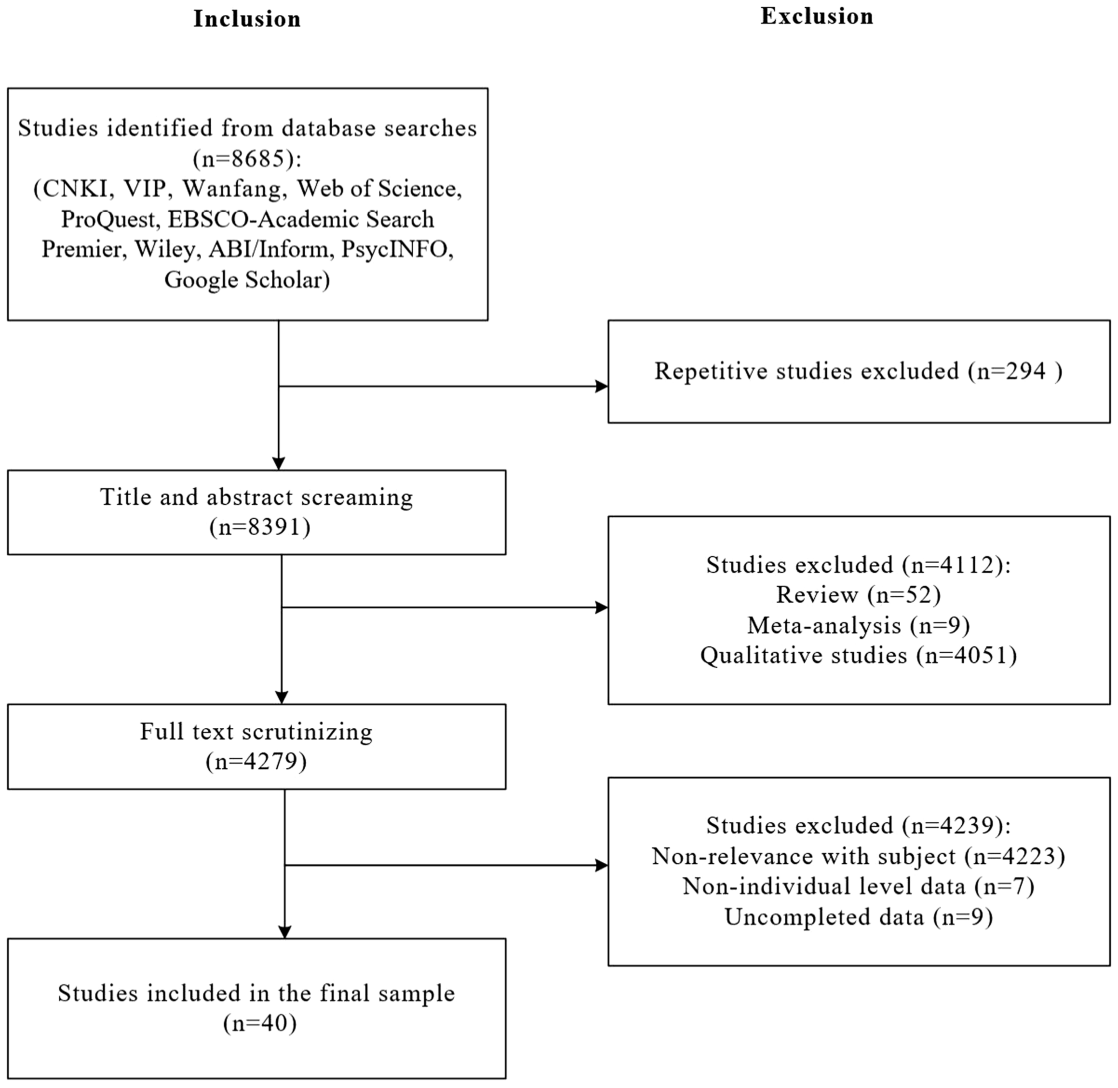
Literature search and inclusion diagram.

Studies included in this meta-analysis had to meet the following criteria: First, studies should report the sample size and r , or t value, F -value, or $\chi^{2}$ that can be converted into r . Second, the level of analysis should be individual. Third, studies should be carried out in an organizational context. Fourth, studies should report the mean value of time pressure. Finally, 40 studies (24 English articles and 16 Chinese articles) were included. The procedures for inclusion and exclusion are presented in Figure 2.

#### Coding of studies

The articles included in the meta-analysis were coded by following the steps recommended by Lipsey and Wilson (2001). We coded (1) author, (2) year of publication, (3) sample size, (4) original correlation coefficient r , (5) mean value of time pressure, and (6) time pressure’s Cronbach’s α and innovation performance’s Cronbach’s α . In addition, according to existing studies (Madigan and Curran, 2021; Zhang et al., 2021), in order to reduce measurement error, the original correlation coefficient *r* in the literature was transformed into the real correlation coefficient by the calculation formula $\frac{r}{\sqrt{\alpha_{x}\alpha_{y}}}$ . In the formula, $\alpha_{x}$ and $\alpha_{y}$ are the α reliability coefficients of independent variable *X* and dependent variable *Y*, respectively. For the articles with missing reliability coefficients, the weighted average reliability was used as the approximate reliability estimator (Joshi et al., 2015). The coding information is shown in Table 1.

**Table 1:** Sample information in Study 1.

References	*n*	*r*	Mean value of time pressure	Cronbach’s α		*ρ*
				Time pressure	Innovation performance	
Adler and Koch (2017)	780	0.190	3.060	0.790	0.950	0.219
Aleksic et al. (2017)	251	0.150	3.147	0.850	0.960	0.166
Amabile et al. (2002-a)	177	−0.020	3.580	0.820	0.810	−0.025
Amabile et al. (2002-b)	177	−0.060	3.227	0.770	0.810	−0.076
Baer and Oldham (2006)	170	−0.240	2.720	0.760	0.980	−0.278
Binnewies and Wornlein (2011-a)	90	0.260	2.390	0.930	0.900	0.284
Binnewies and Wornlein (2011-b)	326	0.210	2.390	0.930	0.900	0.230
Boogerd et al. (2015)	192	0.360	3.770	0.800	0.880	0.429
Bormann (2020)	1,156	−0.020	3.650	0.740	0.780	−0.026
Breevaart and Zacher (2019)	381	0.070	2.460	0.820	0.856	0.084
Chang and Chen (2013)	344	−0.140	3.240	0.660	0.870	−0.185
Chen et al. (2015)	344	−0.140	3.240	0.660	0.870	−0.185
Chen et al. (2017)	379	−0.158	2.701	0.826	0.786	−0.196
Cho et al. (2011-a)	199	0.320	3.620	0.920	0.900	0.352
Cho et al. (2011-b)	199	0.340	3.620	0.920	0.870	0.380
Deng (2009)	239	−0.452	3.052	0.820	0.887	−0.530
Du (2021-a)	78	−0.275	2.317	0.820	0.856	−0.328
Du (2021-b)	78	0.105	2.317	0.820	0.856	0.125
Fan et al. (2020)	413	0.443	3.547	0.884	0.895	0.498
Hsu and Fan (2010-a)	1703	−0.010	3.336	0.820	0.800	−0.012
Hsu and Fan (2010-b)	306	0.062	3.456	0.720	0.860	0.079
Jiang et al. (2019)	325	−0.010	3.650	0.877	0.846	−0.012
Li (2013)	298	0.298	3.356	0.794	0.750	0.386
Lin and Ding (2019)	226	−0.394	3.096	0.908	0.886	−0.439
Maqbool et al. (2019)	608	−0.056	3.207	0.880	0.920	−0.062
McKay (2018-a)	147	−0.200	2.473	0.870	0.870	−0.230
McKay (2018-b)	147	−0.230	2.473	0.870	0.910	−0.258
Michael and Fan (2008)	187	−0.040	3.624	0.820	0.840	−0.048
Moon et al. (2016)	181	0.340	3.280	0.890	0.900	0.380
Naotunna and Zhou (2021)	218	−0.430	3.467	0.960	0.790	−0.494
Noefer et al. (2009-a)	81	0.310	3.230	0.850	0.930	0.349
Noefer et al. (2009-b)	81	0.340	3.230	0.850	0.850	0.400
Ohly et al. (2006)	278	0.180	2.480	0.880	0.920	0.200
Ohly and Fritz (2010)	149	0.180	2.640	0.900	0.960	0.194
Rostami et al. (2019)	216	0.150	3.507	0.820	0.856	0.179
Sijbom et al. (2018)	181	−0.180	2.693	0.850	0.970	−0.198
Song et al. (2019-a)	258	0.470	3.410	0.885	0.922	0.520
Song et al. (2020-a)	366	0.500	3.330	0.615	0.646	0.793
Song et al. (2019-b)	258	−0.244	3.410	0.885	0.908	−0.272
Song et al. (2020-b)	366	−0.215	3.330	0.615	0.681	−0.332
Tong (2016)	274	0.030	3.112	0.850	0.970	0.033
Wang et al. (2019)	289	0.106	3.255	0.788	0.820	0.132
Wang and Wang (2012)	470	−0.050	3.200	0.820	0.856	−0.060
Wang et al. (2021)	364	0.332	3.810	0.820	0.856	0.396
Wang (2020)	265	0.025	3.397	0.882	0.897	0.028
Wu et al. (2014)	179	0.260	3.793	0.820	0.930	0.298
Yao et al. (2020-a)	485	0.472	3.731	0.905	0.860	0.535
Yao et al. (2020-b)	485	0.497	3.679	0.929	0.860	0.556
Yin (2020)	184	0.545	2.734	0.846	0.958	0.605
Zhang et al. (2021)	203	0.060	3.040	0.790	0.890	0.072

a and b are used to distinguish multiple independent samples from the same study; *r* represents original correlation coefficient; ρ presents real correlation coefficient.

#### Data analysis

CMA 3.0 was used to analyze the data. We followed existing studies (Zhao et al., 2021) and used a weighted least squared (WLS) regression analysis to test the J-shaped effect of time pressure on innovation performance. In this analysis, the mean value of time pressure is the independent variable and the correlation coefficient between time pressure and innovation performance is the dependent variable. According to the WLS model results, if the regression coefficient is positive, it suggests that the correlation between time pressure and innovation increased as the mean level of time pressure increased. Further, if the overall effect size (correlation between time pressure and innovation performance) is positive and the 95% CI does not include 0, it means that there is a significant J-shaped relationship between time pressure and innovation performance.

### Results

#### Publication bias

**Figure 3:**
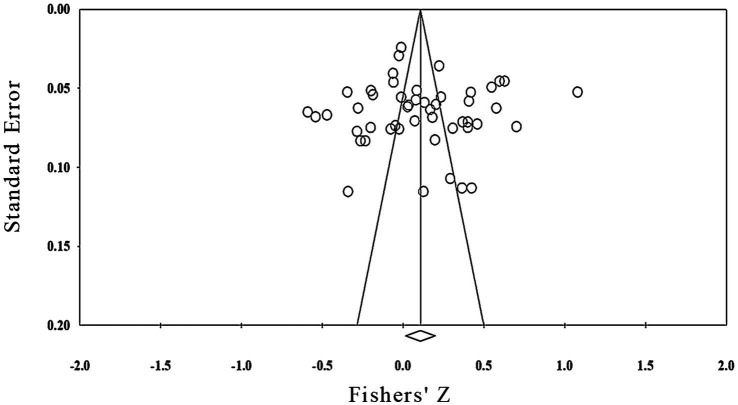
Funnel plot.

**Figure 4:**
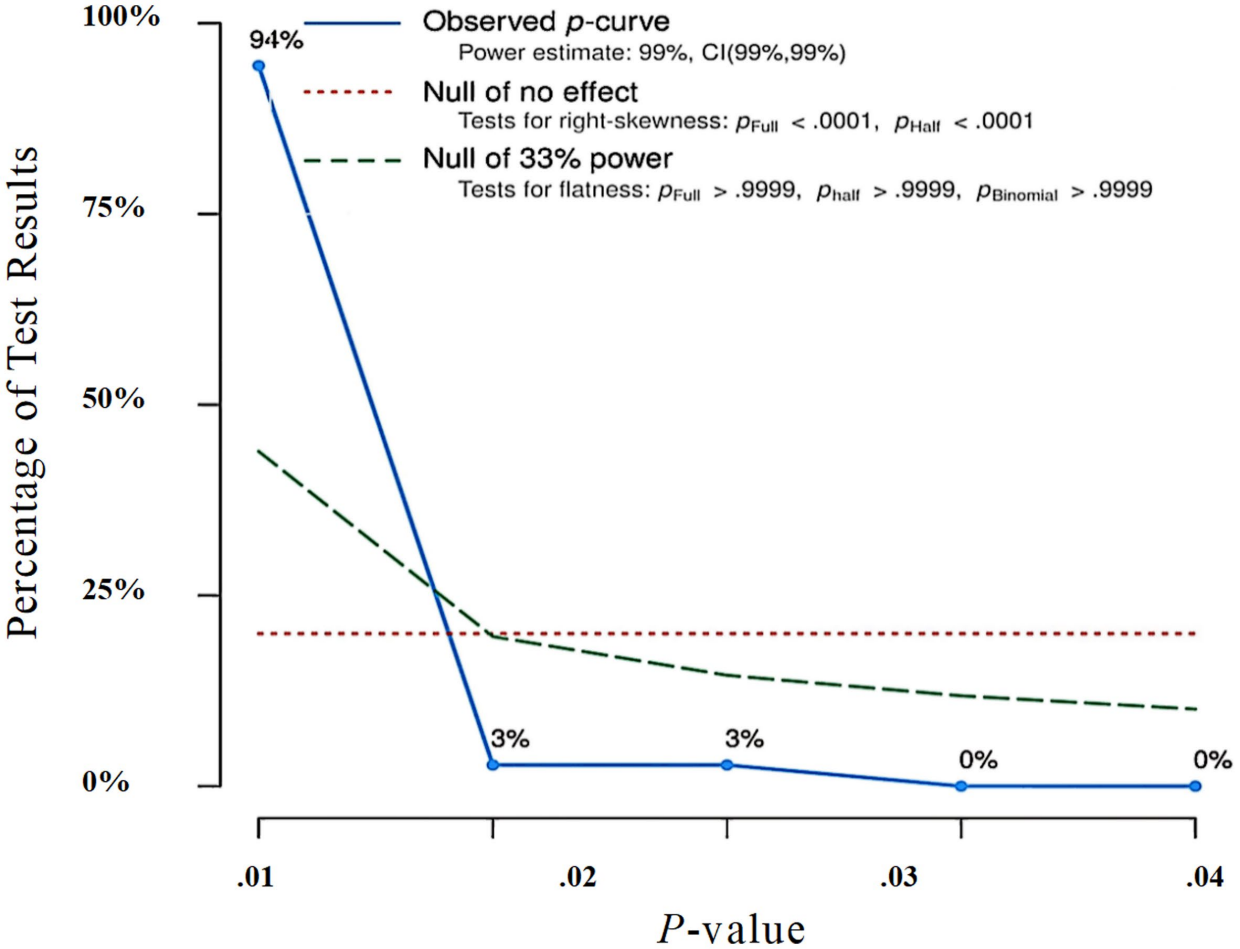
Results of the p-curve analysis.

First, the meta-analysis was tested by funnel plot for publication bias. As shown in Figure 3, the funnel plot is obviously symmetrical, indicating that publication bias was not found. In addition, Egger’s test (*Intercept* = 0.333, *p* = 0.894 > 0.050) and Begg’s rank correlation test (*Z* = 0.134, *p* = 0.894) also showed there was no significant bias. Moreover, as shown in Figure 4, the distribution of *p* values was significantly right-skewed (Binomial test: *p* < 0.0001, Continuous test: *Z* = −24.84, *p* < 0.0001), and all 36 *p* values were lower than 0.025, indicating that there was no significant publication bias for the studies included in the meta-analysis.

#### Model selection

The *Q* test result showed significant heterogeneity in the effect value of the relationship between time pressure and innovation performance (*Q* = 1640.313，*p* < 0.001). In addition, *I^2^* was 97.013%, that is, the true variation of the effect size of the relationship between time pressure and innovation performance accounted for 97.013% of the total variation, which was higher than the high heterogeneity standard of 75%. These results indicate that the random effect model is suitable for estimating the meta-analysis results.

#### Hypotheses test

**Figure 5:**
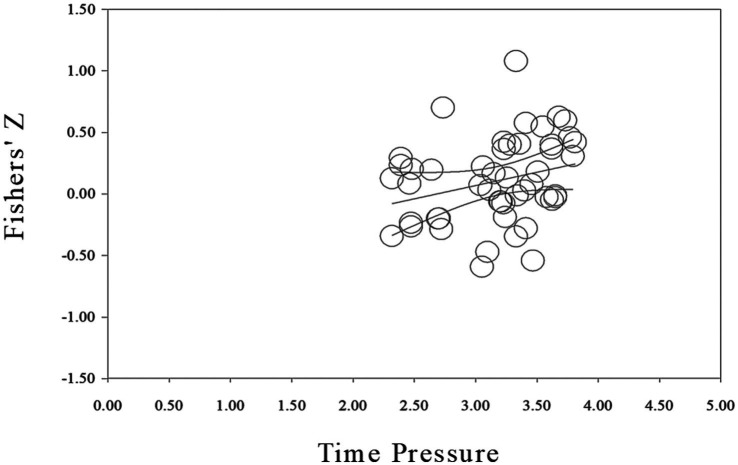
Scatter plot of meta-regression analysis.

Using the mean value of time pressure as the independent variable and the correlation coefficient between time pressure and innovation performance as the dependent variable, the meta-regression analysis showed that the meta-regression coefficient was significantly positive (*b* = 0.216, 95%CI = [0.001, 0.431]), indicating that the effect of the relationship between time pressure and innovation performance gradually increased with increasing time pressure. That is, there was a U-shaped relationship between time pressure and innovation performance. Furthermore, in order to determine whether the U-shaped relationship was a left branch of the U-shape, a right branch of the U-shape (in other words, a J-shape), or completely U-shaped, the overall linear correlation effect between time pressure and innovation performance was analyzed. The results showed that the overall linear correlation coefficient between time pressure and innovation performance was 0.105, 95% confidence interval = [0.014, 0.195], indicating that there was no turning point in the U-shaped relationship between time pressure and innovation performance, presenting a right branch of the U-shape (J-shape). Specifically, as shown in Figure 5, the effect value of the relationship between time pressure and innovation performance was not constant with increasing time pressure, but shows a trend of gradually increasing positive correlation. That is, time pressure had a J-shaped effect on innovation performance. Hypothesis 1 was supported.

## Study 2

### Materials and methods

#### Data collection

To examine the mechanism underlying the J-shaped effect of time pressure on innovation performance, we conducted an online questionnaire survey at two points in time using a convenience sampling approach. Participants were on-the-job MBA students from a university in northern China. A total of 700 participants were invited and 672 participants agreed to participate in the surveys. All participants were full-time employees from various manufacturing and service-sector firms operating in China. They were informed that their identities and responses were anonymous and they could stop answering midway if they felt uncomfortable. Data about demographic variables (age, gender, educational level, and organizational tenure), as well as time pressure and supervisor developmental feedback, were collected in the first online questionnaire. A month later, the second online questionnaire link was sent to employees to measure learning behavior and innovation performance. Finally, 27 participants were excluded from analyses due to missing data, and the final sample included 645 employees. Cook’s distance was applied to identify the outliers. The maximum Cook’s distance was <0.5, indicating that no outliers existed in these data (Zhao et al., 2022). Overall, 249 of the participants were males and 396 were females. The average age was 28.338 years and the average organizational tenure was 4.147 years. Regarding educational level, 103 participants had a junior college degree or below (15.3%), 297 participants had a bachelor’s degree (44.2%), and 272 participants had a master’s degree or above (40.5%).

#### Measurement

Time pressure was measured with a scale developed by Amabile et al. (1996). The scale contained five items, such as “I feel the work time is very urgent.” The Cronbach’s α coefficient was 0.855.

Innovation performance was measured with a scale developed by Scott and Bruce (1994). The scale contained six items, such as “I generate new ideas and creativity in my work.” The Cronbach’s α coefficient of the scale was 0.815.

Learning behavior was measured with a scale developed by Bezuijen et al. (2010). The scale contained eight items, such as “I actively look for methods to improve my work.” The Cronbach’s α coefficient of the scale was 0.814.

Supervisor developmental feedback was measured with a scale developed by Zhou (2003). The scale contained three items, such as “While giving me feedback, my supervisor focuses on helping me to learn and improve.” The Cronbach’s α coefficient of the scale was 0.802.

##### Control variables

Existing studies found that age, gender, educational level, and organizational tenure were significantly related with innovation performance (Fischer et al., 2019; Rangus and Cerne, 2019; He et al., 2020; Dou et al., 2022; Zhong et al., 2022) and time pressure (Skerlavaj et al., 2018; Urbach and Weigelt, 2019). In order to make the effect of time pressure on innovation performance estimated more precisely, we included these variables as control variables, including employee’s age (Mean = 28.338, SD = 3.096, range from 21 to 50 years), gender (0 = male, 1 = female), educational level (1 = junior college degree or below, 2 = bachelor’s degree, and 3 = master’s degree or above), and organizational tenure, which was measured as the number of working years spent in the current organization (Mean = 4.15, SD = 2.42, range from 1 to 20 years).

#### Data analysis

Mplus 7.4 and Medcurve for SPSS 24.0 were used to analyze the data. Firstly, Mplus 7.4 was used to test for common method bias. Secondly, we used SPSS 24.0 to run the multicollinearity test and a principal component analysis. Cronbach’s value and composite reliability were used to evaluate the internal consistency of each variable. Convergent validity was examined with average variance extracted, and discriminant validity was measured by using cross-loadings between all variables. Thirdly, the descriptive statistics and correlations among the variables were examined by using SPSS 24.0. Finally, Medcurve for SPSS 24.0 was applied to test the mediating role of learning behavior and the moderating role of supervisor developmental feedback.

### Results

#### Common method bias analysis and multicollinearity test

Harman’s single-factor test was performed to check common method bias. The results showed that the variation explained by the first factor was 27.966%, which is less than the critical value of 40%, indicating that the effect of common method bias was not a major problem in this study. Further, we performed a confirmatory factor analysis (CFA); as shown in Table 2, the four-factor model obtained a good fit (*χ^2^* = 353.775, *df* = 203, *p* < 0.001; CFI = 0.964, SRMR = 0.043). The model fits were better than other models, indicating that there was not a serious problem with common method bias. Additionally, before conducting data analysis, it is also necessary to test multicollinearity using VIF. If the VIF of the independent variables is greater than 5, the model is considered to have severe multicollinearity (Akinwande et al., 2015). In this study, the VIFs of the variables ranged from 1.013 to 2.199, so there was no significant multicollinearity.

**Table 2:** Results of confirmatory factor analysis in Study 2.

Model	Factor	χ^2^	df	χ^2^/df	TLI	CFI	RMSEA	SRMR
Four-factor model	TP， LB， IP， SDF	353.775	203	1.743	0.959	0.964	0.034	0.043
Three-factor model	TP + LB， IP， SDF	1489.330	206	7.230	0.652	0.690	0.098	0.112
Two-factor model	TP + LB + IP，SDF	1801.817	208	8.663	0.572	0.615	0.109	0.119
One-factor model	TP + LB + IP+ SDF	2180.726	209	10.434	0.473	0.523	0.121	0.126

TP, time pressure; LB, learning behavior; IP, innovation performance; and SDF, supervisor developmental feedback.

#### Reliability and validity analysis

Fornell and Larcker (1981) and Hair et al. (2010) recommend a confidence level of 0.7 or higher to meet the criterion of internal consistency. As shown in Table 3, the Cronbach’s value and composite reliability of each variable were all greater than the recommended values, which indicates good internal consistency in this study. In addition, as shown in Table 3, the average extraction variance (AVE) value for learning behavior was 0.443 > 0.4, and AVE values for other variables were > 0.5, so the convergent validity of the measurement scale was good. Meanwhile, the square root of AVE for each variable was larger than its correlation coefficient with other variables, indicating that the discriminant validity of the scale was good. Finally, as shown in Table 4, the comparison of the factor loadings and cross-loadings for each scale item shows that the factor loadings for each indicator of the specified construct are higher than the loadings on any other construct, indicating that each construct in this study is a unidimensional measure with high internal consistency and reasonable discriminant validity.

**Table 3:** Reliability and validity analysis results in Study 2.

Variable	KMO	CR	AVE	Cronbach’s α
Time pressure	0.844	0.897	0.636	0.855
Learning behavior	0.882	0.862	0.440	0.814
Innovation performance	0.863	0.867	0.521	0.815
Supervisor developmental feedback	0.707	0.884	0.717	0.802

**Table 4:** Results of factor loadings and cross-loadings in Study 2.

Variable	TP	LB	IP	SDF
TP1	**0.769**	0.037	0.094	−0.014
TP2	**0.799**	0.097	0.107	−0.031
TP3	**0.831**	0.052	0.071	0.039
TP4	**0.762**	−0.092	0.032	0.084
TP5	**0.823**	0.047	0.102	0.062
LB1	0.024	**0.701**	0.132	0.041
LB2	−0.025	**0.728**	0.086	0.010
LB3	0.039	**0.645**	0.059	0.129
LB4	0.049	**0.689**	0.272	0.052
LB5	0.072	**0.539**	0.294	0.210
LB6	0.072	**0.624**	0.275	0.014
LB7	0.033	**0.703**	0.210	0.050
LB8	−0.039	**0.661**	0.118	0.014
IP1	0.093	0.245	**0.695**	0.090
IP2	0.112	0.155	**0.726**	0.161
IP3	0.094	0.127	**0.712**	0.249
IP4	0.097	0.196	**0.695**	0.111
IP5	0.027	0.194	**0.726**	0.081
IP6	0.053	0.199	**0.774**	0.170
SDF1	0.101	0.066	0.175	**0.844**
SDF2	0.011	0.070	0.211	**0.830**
SDF3	−0.007	0.134	0.282	**0.866**

The bold values are standardized factor loadings and other values are cross-loadings for each construct.

#### Descriptive analysis and correlation analysis

The mean value, standard deviation, and correlation coefficients of all variables are shown in Table 5. As seen from the results, time pressure was positively correlated with learning behavior (*r* = 0.105, *p* = 0.007) and innovation performance (*r* = 0.221, *p* = 0.000). Learning behavior was positively correlated with innovation performance (*r* = 0.518, *p* = 0.000), which was basically consistent with the expectation of the theoretical hypothesis.

**Table 5:** Mean value, standard deviation, and correlation coefficient of variables in Study 2.

Variable	Mean	SD	1	2	3	4	5	6	7	8
1. Gender	1.614	0.487	1							
2. Educational level	1.992	0.461	−0.027	1						
3. Age	28.338	3.096	−0.094^*^	0.110^**^	1					
4. Organizational tenure	4.147	2.417	−0.061	0.073	0.642^***^	1				
5. Time pressure	3.194	0.901	−0.084^*^	0.007	0.041	0.080^*^	1			
6. Learning behavior	3.910	0.635	−0.055	0.057	0.100^*^	0.093^*^	0.105^**^	1		
7. Innovation performance	3.631	0.716	−0.046	0.093^*^	0.134^**^	0.169^***^	0.221^***^	0.518^***^	1	
8. Supervisor developmental feedback	3.316	0.658	−0.031	0.020	0.155^***^	0.158^***^	0.109^***^	0.266^***^	0.464^***^	1

^***^p < 0.001; ^**^p < 0.01; and ^*^p < 0.05.

#### Hypotheses tests

**Figure 6:**
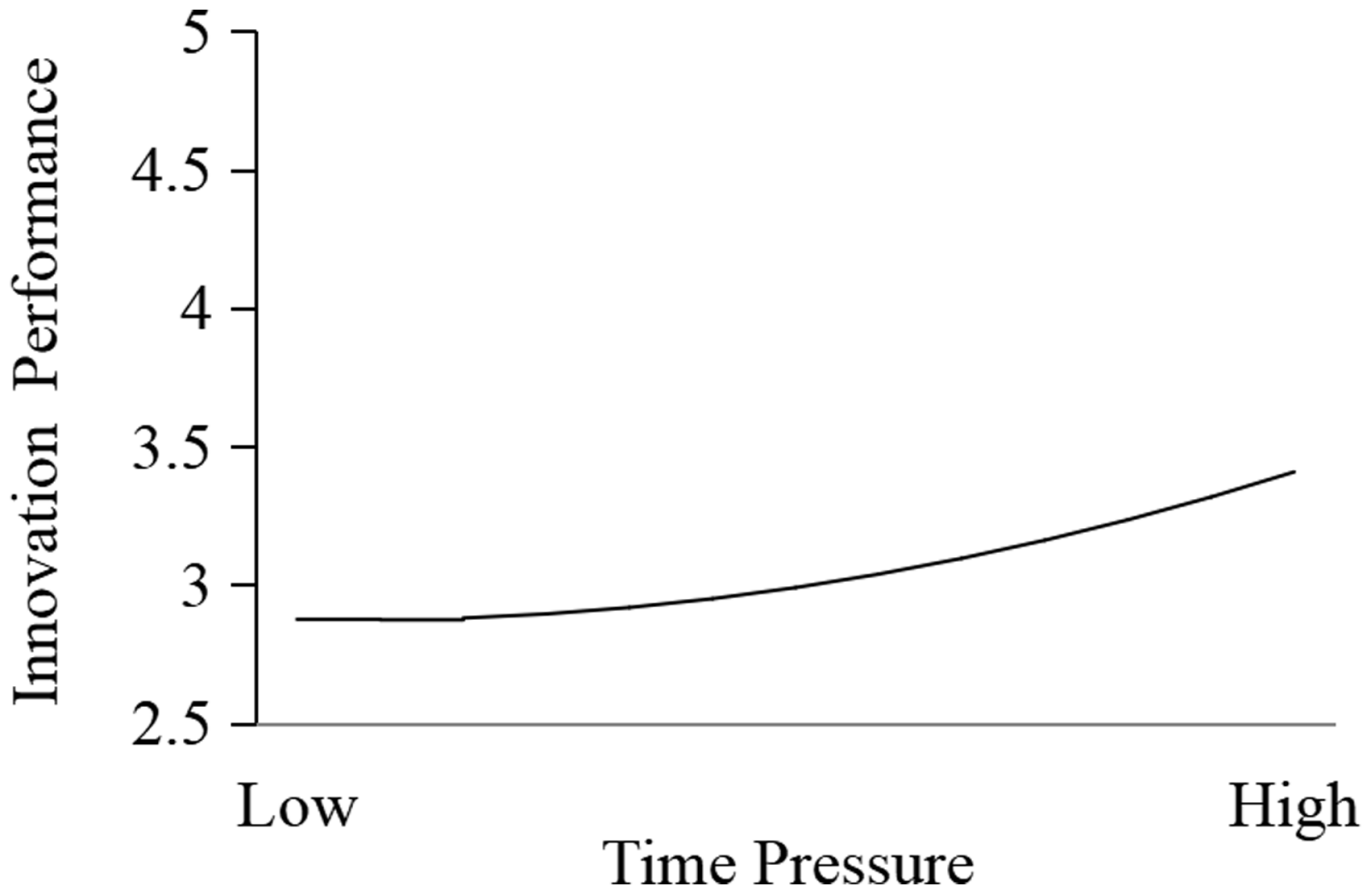
J-shaped effect of time pressure on innovation performance.

Following existing studies (Yucel et al., 2014; Lam et al., 2015; Luu and Ngo, 2019), we tested the J-shaped effect of time pressure on innovation performance. As shown in Model 1 of Table 6, the results showed that time pressure (*b* = 0.197, *p* = 0.000) and time pressure^2^ (*b* = 0.083, *p* = 0.004) both had a significant positive effect on innovation performance. These results indicate that time pressure had a J-shaped effect on innovation performance. As shown in Figure 6, the effect of time pressure on innovation performance increased as the mean value of time pressure increased. Furthermore, referring to existing studies (Haans et al., 2016), the turning point of the J shape (*X* = -*b*/2*a*) was 2.007. Subgroup analysis results showed that the effect of time pressure on innovation performance was not significant (*b* = −0.091, *p* = 0.758) when the time pressure level was lower (*X* < 2.007), but was significantly positive (*b* = 0.241, *p* = 0.000) when the time pressure level was higher (*X* > 2.007). Hypothesis 1 was supported.

**Table 6:** Analyses predicting learning behavior and innovation performance in Study 2.

Variable	Innovation performance				Learning behavior			
	Model 1		Model 2		Model 3		Model 4	
	B	VIF	B	VIF	B	VIF	B	VIF
Gender	−0.020	**1.016**	0.006	1.017	−0.048	**1.016**	−0.041	1.016
Educational level	0.118^*^	**1.013**	0.085	1.015	0.060	**1.013**	0.059	1.030
Age	0.008	**1.722**	0.001	1.726	0.012	**1.722**	0.008	1.731
Organizational tenure	0.034^*^	**1.714**	0.029^*^	1.715	0.009	**1.714**	0.004	1.747
Time pressure	0.197^***^	**1.142**	0.149^***^	**1.158**	0.088^**^	**1.142**	0.058^*^	1.169
Time pressure ^2^	0.083^**^	**1.130**	0.054^*^	**1.138**	0.053^*^	**1.130**	0.025	1.160
Supervisor developmental feedback							0.118^***^	1.904
Time pressure×supervisor developmental feedback							0.121^**^	1.266
Time pressure ^2^ × supervisor developmental feedback							0.112^***^	2.199
Learning behavior			0.546^***^	**1.032**				
Intercept	2.989^***^		1.103^**^		3.439^***^		3.580^***^	
*R^2^*	0.091		0.317		0.031		0.109	
*F*	10.693^***^		42.254^***^		3.382^**^		8.626^***^	

^***^p < 0.001; ^**^p < 0.01; and ^*^p < 0.05.

**Figure 7:**
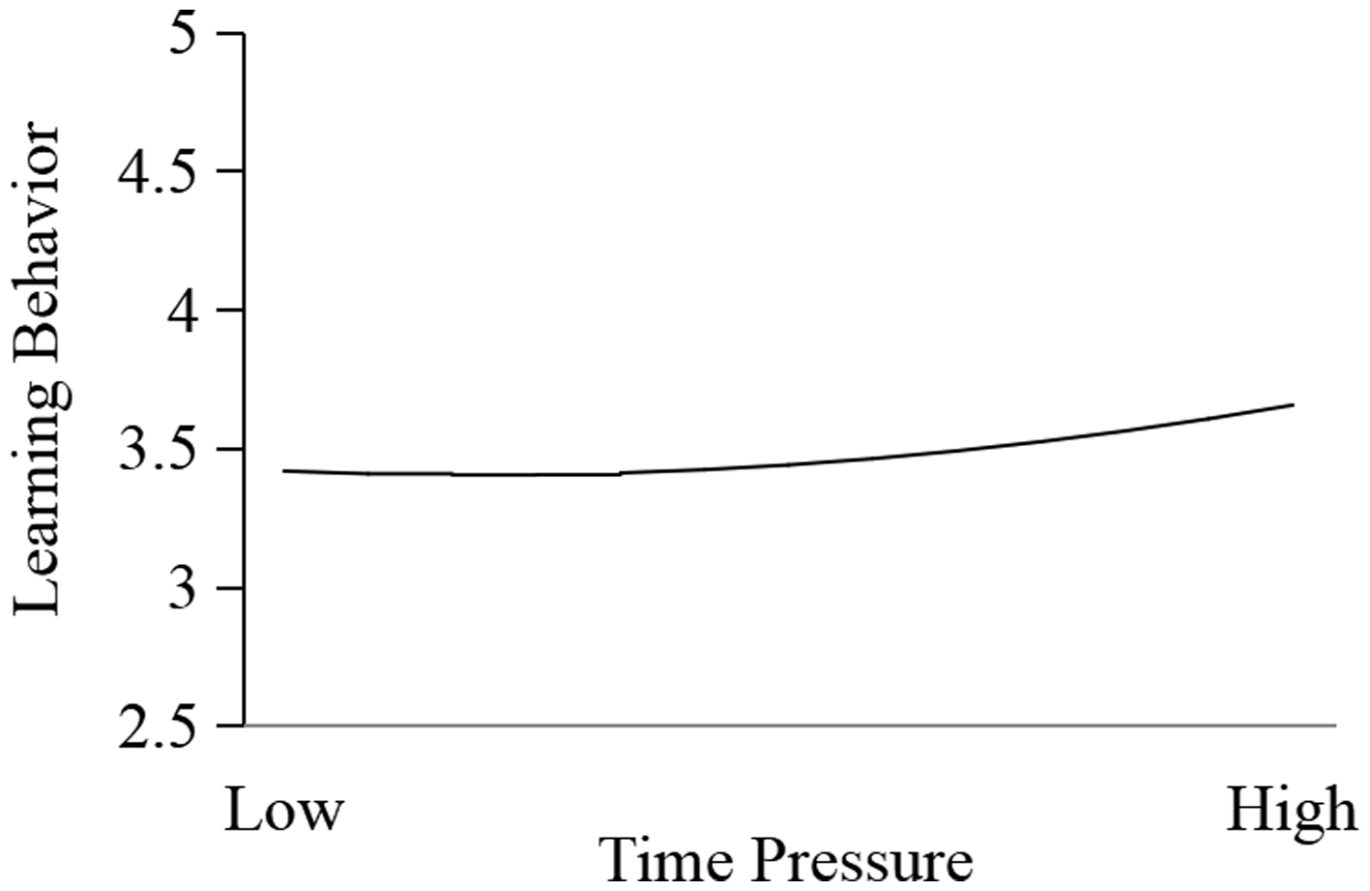
J-shaped effect of time pressure on learning behavior.

Secondly, we tested the J-shaped effect of time pressure on learning behavior. As shown in Model 3 of Table 6, the results showed that time pressure (*b* = 0.088, *p* = 0.003) and time pressure^2^ (*b* = 0.053, *p* = 0.043) had a significant positive effect on learning behavior. Therefore, time pressure had a J-shaped effect on innovation performance. As shown in Figure 7, the effect of time pressure on learning behavior increased as the mean level of time pressure increased. Furthermore, the turning point of the J shape was 2.319. Subgroup analysis results showed that the effect of time pressure on learning behavior was not significant (*b* = 0.077, *p* = 0.664) when the time pressure level was lower (*X* < 2.319), but was significantly positive (*b* = 0.123, *p* = 0.003) when the time pressure level was higher (*X* > 2.319). Hypothesis 2 was supported.

Thirdly, we tested the effect of learning behavior on innovation performance. After controlling for gender, age, educational level, and organizational tenure, the results showed that the effect of learning behavior on innovation performance was significantly positive (*b* = 0.570, *p* = 0.000). Hypothesis 3 was supported.

Fourthly, we tested the mediating effect of learning behavior in the J-shaped relationship between time pressure and innovation performance. As shown in Model 1 of Table 6, the results showed that time pressure (*b* = 0.197, *p* = 0.000) and time pressure^2^ (*b* = 0.083, *p* = 0.004) significantly impacted innovation performance. Then, learning behavior was added into the model. As shown in Model 2 of Table 6, the results showed that the effects of time pressure (*b* = 0.149, *p* = 0.000) and time pressure^2^ (*b* = 0.054, *p* = 0.032) were still significant, and the effect of learning behavior was also significant (*b* = 0.546, *p* = 0.000). Therefore, learning behavior played a partially mediating role in the J-shaped relationship between time pressure and innovation performance. In addition, the mediating effect of learning behavior was tested again according to procedures proposed by Hayes and Preacher (2010). The Medcurve for SPSS 24.0 was used to conduct 5,000 Bootstrap sampling for the sample, and the mediating effect of learning behavior was estimated under different time pressure levels. The results showed that when the time pressure level was low, the mediating effect value was −0.004 and not significant (95%CI = [−0.066, 0.072]), but when the time pressure level was medium or high, both of the mediating effect values were significantly positive: 0.048 (95%CI = [0.013, 0.088]) and 0.100 (95%CI = [0.012, 0.178]). Hypothesis 4 was supported.

**Figure 8:**
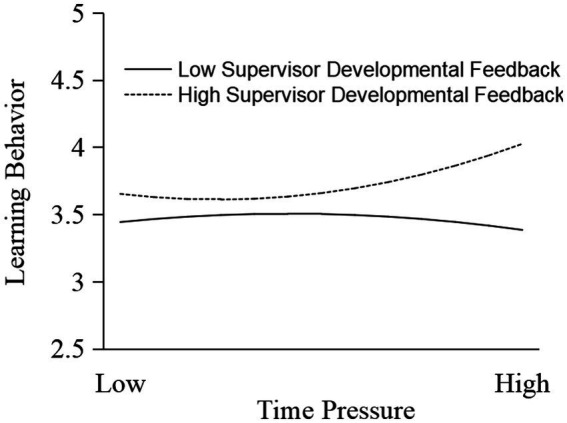
Moderating effect of supervisor developmental feedback.

Fifthly, we tested the moderating effect of supervisor developmental feedback on the relationship between time pressure and learning behavior. As shown in Model 4 of Table 6, the interaction between time pressure^2^ and supervisor developmental feedback had a significant positive effect (*b* = 0.112, *p* = 0.000) on learning behavior, indicating that supervisor developmental feedback had a significant moderating effect on the J-shaped relationship between time pressure and learning behavior. Furthermore, we conducted a subgroup analysis. The results showed that: (1) When the level of supervisor developmental feedback was higher, both the time pressure (*b* = 0.172, *p* = 0.002) and time pressure^2^ (*b* = 0.164, *p* = 0.001) had a significant positive effect on learning behavior. Therefore, time pressure had a J-shaped effect on learning behavior. Before the J-shaped turning point (*X* < 2.804), time pressure had no significant effect on innovation performance (*b* = −0.227, *p* = 0.354), but after that (*X* > 2.804), it had a significant positive effect on innovation performance (*b* = 0.511, *p* = 0.000). (2) When the level of supervisor developmental feedback was lower, both the time pressure (*b* = −0.274, *p* = 0.005) and time pressure^2^ (*b* = −0.198, *p* = 0.017) had a significant negative effect on innovation performance. Therefore, time pressure had an inverted J-shaped effect on learning behavior. Before the inverted J-shaped extreme point (*X* < 2.302), time pressure had no significant effect on learning behavior (*b* = 0.260, *p* = 0.664), but after the turning point (*X* > 2.302), time pressure had a significant negative effect on innovation performance (*b* = −0.301, *p* = 0.026). As shown in Figure 8, time pressure had a J-shaped effect on innovation performance when the level of supervisor developmental feedback was higher, but when the level of supervisor developmental feedback was lower, time pressure had an inverted J-shaped effect on innovation performance. Hypothesis 5 was supported.

Sixthly, we tested the moderating effect of supervisor developmental feedback on the indirect effect of time pressure on innovation performance through learning behavior. Referring to the Monte Carlo method used by Huang et al. (2018), we investigated whether the nonlinear mediating effect was different under different levels of supervisor developmental feedback. The results showed that when the level of supervisor developmental feedback was higher, time pressure had a J-shaped indirect effect on innovation performance through learning behavior; conversely, when the level was lower, time pressure had an inverted J-shaped indirect effect on innovation performance through learning behavior. Specifically, as shown in Table 7, when the level of supervisor developmental feedback was low, the mediating effect of learning behavior was 0.090 and not significant (95%CI = [−0.078, 0.381]) at a low level of time pressure; was −0.083 and significant (95%CI = [−0.182, −0.025]) at a medium level of time pressure; and was −0.256 and significant (95%CI = [−0.570, −0.085]) at a high level of time pressure. On the other hand, when the level of supervisor developmental feedback was high, the mediating effect value of learning behavior was −0.041 and not significant (95%CI = [−0.124, 0.041]) at a low level of time pressure; was 0.072 and significant (95%CI = [0.032, 0.123]) at a medium level of time pressure; and was 0.184 and significantly positive (95%CI = [0.099, 0.336]) at a high level of time pressure. As mentioned above, the mediating effect of learning behavior was significantly different at different levels of supervisor developmental feedback. Hypothesis 6 was supported.

**Table 7:** Moderating effect of supervisor developmental feedback on the mediating effect of learning behavior **(Study 2)**.

Independent variable	Supervisor developmental feedback (low level)		Supervisor developmental feedback (high level)	
	Indirect effect	95%CI	Indirect effect	95%CI
Time pressure (low level)	0.090	−0.078，0.381	−0.041	−0.124，0.041
Time pressure (medium level)	−0.083	−0.182，-0.025	0.072	0.032，0.123
Time pressure (high level)	−0.256	−0.570，-0.085	0.184	0.099，0.336

## Discussion

We explored the J-shaped effect of time pressure on innovation performance and its underlying mechanism. All hypotheses were supported by the findings. The results showed that time pressure had a significant J-shaped effect on innovation performance: that is, time pressure did not significantly promote innovation performance at lower levels of time pressure, but when it increased to medium and higher levels, time pressure significantly improved innovation performance. In addition, the reason why time pressure had a J-shaped effect on innovation performance was revealed by exploring the nonlinear mediating effect of learning behavior. When time pressure increased to a certain level, it would motivate employees’ learning behaviors, and thus significantly promoted innovation performance. Finally, the J-shaped effect of time pressure on innovation performance through learning behavior had boundary conditions. When the level of supervisor developmental feedback was higher, time pressure had a J-shaped effect on innovation performance through learning behavior: that is, when time pressure reached a certain level, it had a positive effect on innovation performance by driving employees to produce learning behavior. On the other hand, when the level of supervisor developmental feedback was lower, time pressure had an inverted J-shaped effect on innovation performance through learning behavior: that is, when time pressure reached a certain level, it had a negative effect on innovation performance by discouraging employees from producing learning behavior.

### Theoretical contributions

Firstly, existing studies mainly analyzed the linear or inverted U-shaped relationship between time pressure and innovation performance (Binnewies and Wornlein, 2011; Breevaart and Zacher, 2019). Although some studies questioned the inverted U-shaped relationship and pointed out that high levels of time pressure may result in more creativity (Aleksic et al., 2017), neither in-depth theoretical analysis nor empirical research had been carried out to test this viewpoint. Compared to previous studies, our study shed light on the J-shaped relationship and the mechanisms underlying it, expanding and deepening understanding of the nonlinear relationship between time pressure and innovation performance. Moreover, some studies called for research to explore more complex nonlinear effects of stress other than the inverted U-shaped effect, such as the J-shaped effect of work stress (Yankelevich et al., 2012; Pindek et al., 2022). Responding to existing research calls, our research results found the J-shaped effect of time pressure on innovation performance and then enriched understanding of the nonlinear effect of work stress.

Secondly, few studies used meta-analysis to explore the relationship between time pressure and innovation performance, although some studies have explored this relationship. We explored the J-shaped relationship between time pressure and innovation performance using a systematic meta-analysis, which allowed the effect of time pressure on innovation performance to be estimated more accurately and reliably. In addition, existing studies did not reach consensus on whether the positive relationship between the time pressure and innovation performance is significant (Adler and Koch, 2017; Rostami et al., 2019). By revealing the J-shaped relationship between the two using a meta-analysis, we also integrated the existing inconsistent research results: we found that whether or not time pressure had a significant positive effect on innovation performance depended on the level of time pressure. When the level of time pressure was lower, the positive effect was not significant, but when it increased to a higher level, the positive effect was significant.

Thirdly, existing studies explored the indirect effect of time pressure on innovation performance through the mediating effect of cognitive and emotional responses; these approaches were based on cognitive appraisal theory, affective event theory, activation theory, etc. (Binnewies and Wornlein, 2011; Adler and Koch, 2017; Naotunna and Zhou, 2021), but they rarely discussed the mediating effect of learning behavior as a possible underlying mechanism. The current study explored the possible mechanism underlying the relationship between time pressure and innovation performance based on the active learning hypothesis, which not only verified the mediating effect of learning behavior, but also revealed the positive and negative mediating effects of learning behavior under different levels of supervisor developmental feedback. The results enriched and deepened the understanding of the mechanism underlying the relationship between time pressure and innovation performance. In addition, few existing empirical studies focused on learning behavior under work stress from a nonlinear perspective, but our research revealed the nonlinear effect of stress on learning behavior in a more detailed and in-depth way by identifying the J-shaped and inverted J-shaped effects of time pressure on learning behavior under different levels of supervisor developmental feedback.

Fourthly, existing studies mainly explored the boundary conditions of the relationship between time pressure and innovation performance from the perspective of individual characteristics (Antwi et al., 2019; Rostami et al., 2019; Bormann, 2020), but rarely discussed the boundary conditions from the perspective of leader behavior. Compared with previous studies, we expanded the existing literature by taking supervisor developmental feedback as a boundary condition. The results showed that supervisor developmental feedback not only moderated the relationship between time pressure and innovation performance, but also was a decisive factor for deciding whether time pressure had a positive or negative effect on innovation performance. The results provide a beneficial supplement for identifying the boundary conditions of the relationship between time pressure and innovation performance. In addition, few studies focused on the moderating effect of supervisor developmental feedback between work stress and learning behavior. By examining the moderating effect of supervisor developmental feedback in the intermediary effect of time pressure on innovation performance through learning behavior, we also expanded understanding of the boundaries of the relationship between time pressure and learning behavior, and advanced the research field of supervisor developmental feedback.

### Practical implications

First of all, managers should recognize employees’ subjective initiative under time pressure, and consider taking advantage of time pressure by setting work time schedules to motivate employees’ work involvement and then improve their innovation performance. But at the same time, given the rules about working hours in the Labor Law, and the too-much-of-a-good-thing effect, we emphasize that organizations should not be too extreme about putting time pressure on employees. Secondly, managers should recognize the important mediating effect of learning behavior in the relationship between time pressure and innovation performance, and take care to provide sufficient learning resources and create a good learning atmosphere so as to help employees engage in more learning behavior under time pressure. Finally, leaders should realize that supervisor developmental feedback is a key factor that can determine whether the effect of time pressure on innovation performance is positive or negative. Leaders should provide more developmental feedback to employees who are under time pressure. In this way, employees will enhance their resources to cope with time pressure, and then generate more learning behaviors to improve innovation performance.

### Limitations and future research

Firstly, we verified the J-shaped nonlinear effect of time pressure on innovation performance based on a meta-analysis. However, the stability and reliability of meta-analysis results depend on the comprehensiveness of the literature. Limited by language, we mainly collected Chinese and English articles. Future studies should include more literature in different languages to improve the reliability and validity of the meta-analysis results. Secondly, we used employees’ self-report to measure innovation performance. Although the results found that there was no common method bias, future research could ask supervisors to evaluate their subordinates’ innovation performance so as to enhance the accuracy of results. Thirdly, we explored the relationship between time pressure and innovation performance from a general perspective. However, previous studies have pointed out that time pressure can be divided into challenge and hindrance time pressure (Chong et al., 2011); innovation performance could be divided into incremental innovation and radical innovation (Sheehan et al., 2021); and learning behavior could also be divided into exploration and exploitation in organizational learning (March, 1991). Thus, future studies can explore the various relationships between different types of time pressure and innovation performance. Finally, previous studies have found that social support in the workplace had an important effect on the relationship between stress and learning behavior (Ouweneel et al., 2009). We mainly discussed the moderating effect of supervisor developmental feedback from the perspective of social support. However, there are many other types of social support. For example, leader-member exchange (LMX) refers to the quality of the supervisor-employee relationship, which is significantly related to supervisor developmental feedback, and directly affects how well subordinates perceive feedback from their superiors (Kim, 2006; Sparr and Sonnentag, 2008). Future studies can analyze the moderating effects of LMX on the relationship between time pressure and learning behavior. In addition, future studies can analyze the moderating effects of co-worker support, such as information sharing and helping behavior from co-workers, in order to reveal more boundaries of the relationship from the perspective of social support.

## Conclusion

This paper aimed to examine how time pressure affects employees’ innovation performance using two studies. The results showed that: (1) Time pressure had a J-shaped effect on innovation performance, in which the effect of time pressure on innovation performance was not significant at the beginning but was significantly positive after reaching the J-shaped critical point; (2) Time pressure had a J-shaped indirect effect on innovation performance through the mediating effect of learning behavior, in which the indirect effect was not significant at the beginning but was significantly positive after reaching the critical point; and (3) The moderating effect of supervisor developmental feedback was significant: when the level of supervisor developmental feedback was high, time pressure had a J-shaped effect on innovation performance through the positive nonlinear mediating effect of learning behavior; but when the level was low, time pressure had an inverted J-shaped effect on innovation performance through the negative nonlinear mediating effect of learning behavior. The findings contribute to answering how and why time pressure had a J-shaped effect on innovation performance, providing important implications to the literature on time pressure. In addition, the findings also provide guidance for organizations and employees about how to cope with time pressure and improve innovation performance.

## Data availability statement

The original contributions presented in the study are included in the article/supplementary material, further inquiries can be directed to the corresponding author.

## Ethics statement

The studies involving human participants were reviewed and approved by the Ethics Committee of Zhengzhou University. Written informed consent for participation was not required for this study in accordance with the national legislation and the institutional requirements.

## Author contributions

HS contributed to the study’s framework construction and data analysis, and wrote the first draft of the manuscript. RG, QZ, and YL were responsible for collecting data, revising the paper, and translating the manuscript. All authors contributed to the article and approved the submitted version.

## Funding

The study was funded by the National Social Science Foundation of China (19CGL026).

## Conflict of interest

The authors declare that the research was conducted in the absence of any commercial or financial relationships that could be construed as a potential conflict of interest.

## Publisher’s note

All claims expressed in this article are solely those of the authors and do not necessarily represent those of their affiliated organizations, or those of the publisher, the editors and the reviewers. Any product that may be evaluated in this article, or claim that may be made by its manufacturer, is not guaranteed or endorsed by the publisher.
